# Ceftaroline in CNS and ocular infections: a case series

**DOI:** 10.1093/jacamr/dlae095

**Published:** 2024-06-17

**Authors:** Emily A Siegrist, Joseph Sassine

**Affiliations:** Department of Pharmacy, OU Health, OU Medical Center, 700 NE 13th St., Oklahoma City, OK 73104, USA; Infectious Diseases Section, Department of Medicine, The University of Oklahoma Health Sciences Center, 800 Stanton L. Young Blvd, Oklahoma City, OK 73104, USA

## Abstract

**Background:**

There are limited data describing outcomes of patients treated with ceftaroline for infections with CNS or ocular involvement.

**Objectives:**

To describe outcomes of patients treated with ceftaroline for methicillin-resistant staphylococcal infections involving the CNS or eye.

**Patients and methods:**

This was a retrospective review of 10 patients at an academic medical centre who received ceftaroline for CNS or ocular infections.

**Results:**

All patients were treated with ceftaroline as part of a combination for salvage therapy. Four patients died, whereas six patients experienced clinical cure. Only one experienced microbiological recurrence.

**Conclusions:**

These preliminary data suggest that ceftaroline may be an option for salvage therapy of severe staphylococcal infections when used in combination.

## Introduction

Ceftaroline was FDA approved in 2010 for the treatment of acute bacterial skin and skin structure infections and community-acquired bacterial pneumonia.^1^ Like other cephalosporins, ceftaroline inhibits cell wall synthesis by binding to PBPs. Unlike other cephalosporins, ceftaroline has expanded activity against MRSA due to its increased affinity for PBP2a.^1^

For CNS infections due to methicillin-resistant staphylococci, guidelines recommend vancomycin with or without the addition of rifampin;^2,3^ however, these guidelines do not take into account more recent literature regarding treatment of complicated MRSA infections. Furthermore, vancomycin achieves low CSF concentrations, particularly in uninflamed meninges.^4^ Guidelines recommend consideration of an alternative agent for staphylococcal species with vancomycin MIC  ≥1 mg/L.^2^ Alternative agents for CNS infections caused by methicillin-resistant staphylococci include rifampin, linezolid, trimethoprim/sulfamethoxazole and daptomycin; however, evidence supporting the efficacy of any of these therapies is limited.^2,3^ Daptomycin use is limited by poor CNS penetration,^5^ and linezolid use by serotonergic drug interactions and haematological toxicity with prolonged use.^6^ Additionally, evidence supporting vancomycin for the treatment of ophthalmic infections is limited to retrospective reviews and case series.^7^ Ceftaroline may be an alternative option in patients with CNS or ocular infections. Previous studies have shown that ceftaroline in combination with daptomycin may improve outcomes in patients with bacteraemia,^8^ Additionally, in some models, ceftaroline has favourable pharmacokinetics in inflamed meninges.^9^

Given the known limitations of currently available therapies, ceftaroline is a promising option for the treatment of CNS and ocular infections. The aim of this study was to describe the use of ceftaroline in combination for the treatment of CNS and ocular infections.

## Patients and methods

This was a retrospective, single-centre case series. Patients who received ceftaroline were identified using the VigiLanz database (VigiLanz Corporation, Minneapolis, MN, USA). Patients admitted from January 2015 through February 2022 were included if they received ceftaroline for a CNS or an ocular infection, if they were ≥18 years of age and if they received ceftaroline for ≥48 h. Patient demographics (i.e. gender, ethnicity, comorbidities, social history), treatment characteristics (i.e. concomitant antibiotics, duration of therapy, source control interventions) and outcomes (i.e. disposition at discharge, mortality) were collected. Susceptibility testing was conducted using overnight broth dilution panels (MicroScan, Beckman Coulter). Descriptive statistics were performed.

## Results

Ninety unique, adult patients who received ceftaroline were identified. Ten patients met the inclusion criteria, of whom four were treated for an ocular infection and six were treated for a CNS infection. Patient demographics, baseline comorbidities and sites of infection are shown in Table 1. Ceftaroline was started at a median of 6 days after diagnosis, in general due to a lack of clinical response or culture clearance and was continued for a median of 11 days (Table 2). All except one patient received vancomycin prior to the initiation of ceftaroline. Ceftaroline was given as salvage therapy in combination with vancomycin, linezolid or daptomycin in all patients. Of the seven patients with persistent bacteraemia at the time of ceftaroline initiation, median time to blood culture clearance was 3 days (range 1–28 days). Five patients received ceftaroline 600 mg q8h (creatinine clearance (CrCl)  > 50 mL/min), two received 400 mg q8h (CrCl 30–50 mL/min) and three received 300 mg q12h (CrCl < 30 mL/min). Ceftaroline was typically discontinued in patients clinically improving on combination therapy after several days.

**Table 1.** Baseline demographics and infection characteristics

Patient	Age, y	Gender	Race	Comorbidities	Substance use	Charlson comorbidity index	Sites of infection	Pitt bacteraemia score	Organism	VAN MIC	Sites of positive culture	Duration of bacteraemia, d	Findings on CNS imaging
1	63	Female	Caucasian	Hypertension, heart disease, chronic lung disease	Tobacco, non-IV drugs	3	Blood, lung, eye	1	MRSA	1	Blood, sputum	4	Ocular infection
2	50	Female	Caucasian	Hypertension, solid malignancy	None	5	Skin and soft tissue, bone, CSF, infected hardware	Not applicable	*Staphylococcus epidermidis*	2	Tissue, CSF, abscess	Not applicable	None
3	33	Male	Caucasian	Uncontrolled diabetes	Tobacco, alcohol	2	Blood, skin and soft tissue, septic thrombophlebitis, lung, eye, pleural fluid	0	MRSA	2	Blood, tissue, vitreous fluid, pleural fluid	12	Ocular infection
4	45	Male	Caucasian	Hypertension, heart disease, CKD/ESRD, chronic lung disease	Tobacco, non-IV drugs	5	Blood, septic thrombophlebitis, brain, spinal cord	2	MRSA	1	Blood, BAL	11	Septic emboli, ventriculitis
5	36	Male	Caucasian	None	Tobacco, IV and non-IV drugs	0	Blood, heart valve, CSF, brain, genitourinary	2	MRSA	2	Blood, urine	4	Septic emboli
6	64	Male	African American	Uncontrolled diabetes, hypertension, CKD/ESRD	None	6	Blood, septic thrombophlebitis, lung, joint, eye, CVC	3	MRSA	1	Blood, tracheal aspirate, BAL, synovial fluid, vitreous fluid	47	None
7	76	Female	Caucasian	Haematological malignancy status post-HCT and CAR T cell therapy, obesity	None	5	Blood, skin and soft tissue, CSF	2	MRSA	1	Blood, tissue, wound	12	Ventriculitis
8	74	Male	Caucasian	Diabetes, hypertension, heart disease, CKD/ESRD, obesity	None	7	Blood, skin and soft tissue, bone, eye	0	MRSA	2	Blood	12	None
9	69	Male	Caucasian	Hypertension, chronic lung disease, autoimmune disease	None	3	Blood, skin and soft tissue, heart valve, lung, brain	3	MRSA	2	Blood, sputum	8	Septic emboli
10	52	Male	Caucasian	Uncontrolled diabetes, hypertension	Tobacco, IV drugs	4	Blood, CSF, brain, CVC	10	MRSA	1	Blood	5	Meningitis, septic emboli, ventriculitis

BAL, bronchoalveolar lavage; CAR, chimeric antigen receptor; CKD/ESRD, chronic kidney disease/end-stage renal disease; CVC, central venous catheter; HCT, hematopoietic cell transplantation; VAN, vancomycin.

**Table 2.** Infection management and patient outcome

Patient	Antibiotics before CPT (d)	CPT DOT	Concomitant antibiotics (d)	Antibiotics after CPT (d)	Inpatient antibiotic DOT	Highest level of care	Survival outcome	Cause of death	LOS, d	Readmission	Microbiological recurrence
1	VAN (6)	8	VAN (7)	VAN (9)	23	ICU	Death during admission	Index infection	22	N/A	None
2	VAN (3), DAP (2)	6	VAN (1)	DAP (7)	16	ICU	Death after discharge	Non-infectious	15	Within 90 d	Within 30 d
3	VAN (4)	21	VAN (1), LZD (19)	VAN (6)	30	ICU	Alive at time of review	N/A	29	Within 30 d	None
4	VAN (9)	20	VAN (19)	VAN (3)	31	ICU	Alive at time of review	N/A	30	None	None
5	None	11	VAN (4), DAP (8)	VAN (4)	15	Floor	Unknown	N/A	14	None	None
6	VAN (19)	27	LZD (2), DAP (27)	LZD (13), DAP (13)	57	ICU	Death during admission	Index infection	57	N/A	None
7	VAN (10), DAP (9)	23	VAN (20), DAP (4)	None	35	Floor	Death after discharge	Unknown	46	None	None
8	VAN (4)	8	DAP (7)	None	11	Floor	Death during admission	Non-infectious	11	N/A	None
9	VAN (3)	11	DAP (10)	None	17	ICU	Death during admission	Index infection	16	N/A	None
10	VAN (3)	4	VAN (1), DAP (2)	None	7	ICU	Death during admission	Index infection	7	N/A	None

CPT, ceftaroline; DAP, daptomycin; DOT, days of therapy; LOS, length of stay; LZD, linezolid; N/A, not applicable; VAN, vancomycin.

All patients with ocular involvement had MRSA bacteraemia, with some additionally having concomitant involvement of distant sites, including skin and soft tissue and lung. Generally, infections were deep-seated and intravascular source was common. Vitreous fluid cultures were positive for MRSA in two (50%) patients, and two (50%) had evidence of ocular infection on CNS imaging. Two patients received repeated intravitreal infections with vancomycin, and one of these additionally required right eye enucleation and left eye pars plana vitrectomy.

Of the six patients with CNS infection, five (83%) had a concomitant bacteraemia, with MRSA being the causative organism. Looking at the entire cohort, duration of bacteraemia ranged from 4 to 47 days. The Pitt bacteraemia score was ≥2 and >3 in six and three patients, respectively, indicating high risk of mortality. CSF analysis was done on three patients. The majority of CNS involvement was diagnosed based on brain imaging. At the time of review, six patients had died, with four deaths being attributed to the index infection. Five patients were successfully treated and discharged, and one patient experienced microbiological recurrence within 30 days (Table 2).

## Discussion

To our knowledge this is the largest case series describing the outcomes of patients treated with ceftaroline for methicillin-resistant staphylococcal infections of the eyes or CNS. Due to the lack of prospective studies, the optimal treatment for CNS or ocular infections due to resistant staphylococci is largely based on expert opinion and retrospective studies.^2,3^ Particularly, the optimal treatment strategy for the patients who respond slowly or fail to respond to vancomycin is unknown and only relies on case reports and clinical judgement. Previous studies have shown that ceftaroline and daptomycin combination therapy may improve outcomes in patients with *Staphylococcus aureus* bacteraemia,^8^ but conclusions cannot be made about efficacy for CNS or ocular infections, as they represent a very small minority of patients.

Although several studies have examined off-label uses of ceftaroline and included small numbers of patients with meningitis, these studies did not describe characteristics and outcomes of these patients in detail.^10,11^ Balouch *et al.*^12^ described a patient successfully treated for MRSA meningitis with a combination of ceftaroline and rifampin. In a case series of five patients with Gram-positive meningitis, one patient was included who had MSSA meningitis.^13^ This patient was successfully treated with ceftaroline monotherapy after susceptibility testing confirmed a MIC < 0.5 mg/L. We identified only one case report of a patient with endogenous endophthalmitis treated with ceftaroline.^14^

Four patients (40%) in our cohort died due to the index infection that was being treated with ceftaroline. Mortality in patients with endogenous endophthalmitis is poorly described; however, CNS involvement with *S. aureus* bacteraemia was associated with significant in-hospital mortality of 25%–56% in previous studies,^15^ and CNS involvement was an independent predictor of mortality in patients with *S. aureus* bacteraemia with an OR of 12.9 (95% CI: 1.1–152.9) in one retrospective study.^15^ Further, the median duration of bacteraemia in our study was 7 days, and the median Pitt bacteraemia score was 2, both of which are indicators of increased risk for clinical failure.^16,17^ The mortality rate of patients treated with ceftaroline in this study is consistent with previous literature and shows that this cohort of patients likely represents a subset of patients who were already at elevated risk of mortality due to the severity of the infection.

Half of the patients in our case series had vancomycin MICs of 2 mg/L, for which AUC/MIC targets may be difficult to achieve, particularly in difficult-to-reach sites like the CSF or vitreous fluid. Due to the retrospective nature of this study, we were not able to measure ceftaroline concentrations in the CSF or vitreous fluid, nor were we able to confirm MICs using broth microdilution or Etest. CSF concentrations of ceftaroline have been reported as only 2%–9% of plasma concentration in uninflamed meninges, and peak CSF concentrations after a single 600 mg dose were estimated to be below the MIC for most pathogens, at only 0.22 mg/L,^18–20^ suggesting that ceftaroline may not be ideal for the treatment of CNS infections in which the meninges are not inflamed. However, a recent study using Monte Carlo simulation evaluated ceftaroline for the treatment of MRSA meningitis by modelling various levels of meningeal inflammation, estimated by CSF glucose concentration. This study found that the PTA—defined as free time above MIC of 34.7%—in inflamed meninges was 99.8% if thrice daily ceftaroline dosing was used.^9^ Thrice daily dosing was the most common dosing frequency in this cohort, likely translating to a high PTA in patients with inflamed meninges.

This study was limited by its retrospective design and the absence of a comparator arm, thus no firm conclusions about the efficacy of ceftaroline can be determined from this study. Additionally, as ceftaroline was not used as monotherapy, the efficacy of ceftaroline alone cannot be ascertained and conclusions about mortality rate of patients treated with ceftaroline cannot be determined. This cohort of patients had multisite, deep-seated infections and clinical improvement with ceftaroline may have been due to ceftaroline’s activity in sites other than the CNS and eyes. Further studies are needed to determine the role of ceftaroline monotherapy and to determine the best partner agent for combination therapy.
